# Distinct Molecular Mechanisms Underlie Modulation of Seeded α-Synuclein Aggregation and Toxicity by Salvianolic Acid B and Dihydromyricetin

**DOI:** 10.3390/ijms27093843

**Published:** 2026-04-26

**Authors:** Nishant N. Vaikath, Iman W. Achkar, Indulekha P. Sudhakaran, Ilham Y. Abdi, Janarthanan Ponraj, Omar M. A. El-Agnaf

**Affiliations:** 1 Neurological Disorders Research Center, Qatar Biomedical Research Institute (QBRI), Hamad Bin Khalifa University (HBKU), Education City, Qatar Foundation, P.O. Box 5825, Doha 34110, Qatar; 2 Materials Core Laboratoires (MCL), Hamad Bin Khalifa University (HBKU), Doha 34110, Qatar

**Keywords:** α-synuclein, aggregation, polyphenolic inhibitors, Parkinson’s disease

## Abstract

Aggregation and seeded propagation of α-synuclein (α-syn) are central to the pathogenesis of Parkinson’s disease and related synucleionopathies. Modulation of seeded aggregation and amplification of pathological α-syn species represents a promising strategy for limiting disease progression. Here, we investigated the effects of naturally derived polyphenolic compounds on α-syn fibrillation, seeded aggregation, and associated cytotoxicity. Among the compounds examined, salvianolic acid B and dihydromyricetin exhibited significant inhibitory effects on α-syn aggregation. Biochemical and biophysical analyses using Thioflavin-T fluorescence, Congo Red binding, and transmission electron microscopy demonstrated that both compounds inhibited fibril formation and altered fibril morphology. Notably, dihydromyricetin efficiently disaggregated preformed fibrils and suppressed seeded fibril elongation, whereas salvianolic acid B primarily delayed aggregation kinetics. Both compounds significantly reduced α-syn-induced cytotoxicity in BE(2)-M17 cells. These findings demonstrate that salvianolic acid B and dihydromyricetin differentially modulate key steps in the α-syn aggregation pathway and reduce associated cellular toxicity. Collectively, these results provide mechanistic insight into the modulation of seeded α-syn aggregation and identify salvianolic acid B and dihydromyricetin as effective modulators of pathological α-syn assembly.

## 1. Introduction

Parkinson’s disease (PD) is a progressive, typically late-onset neurodegenerative disorder characterized clinically by motor and non-motor symptoms and pathologically by the selective degeneration of dopaminergic neurons in the substantia nigra pars compacta [1,2]. The loss of these neurons leads to striatal dopamine deficiency and underlies many of the hallmark motor manifestations of the disease. A defining neuropathological feature of PD is the presence of intracellular proteinaceous inclusions, known as Lewy bodies (LBs) and Lewy neurites (LNs), within vulnerable neuronal populations [3].

Alpha-synuclein (α-syn), a presynaptic neuronal protein, is the principal component of these inclusions and plays a central role in PD pathogenesis [3,4,5]. Beyond PD, pathological aggregation of α-syn is a shared feature of several related neurodegenerative disorders, including dementia with Lewy bodies (DLB) and multiple system atrophy (MSA), collectively referred to as synucleinopathies [5,6,7]. Genetic, biochemical, pathological, and experimental studies strongly support the causal involvement of α-syn misfolding and aggregation in disease initiation and progression [7,8,9].

Under physiological conditions, α-syn exists predominantly as a soluble, intrinsically disordered protein. However, under pathological conditions, it undergoes a conformational transition leading to oligomerization and fibril formation [10]. In vitro studies have demonstrated that α-syn fibrillation follows a nucleation-dependent process, consisting of an initial lag phase, a rapid elongation phase, and a final steady-state equilibrium [11,12]. Increasing evidence suggests that oligomeric and fibrillar α-syn species can act as seeds, promoting templated misfolding of endogenous α-syn and facilitating the cell-to-cell propagation of pathology across anatomically connected brain regions [4,13,14,15,16].

This prion-like propagation of α-syn pathology is now widely recognized as a key mechanism driving disease progression in synucleinopathies [17]. As a result, therapeutic strategies aimed solely at inhibiting de novo fibril formation may be insufficient. Instead, targeting seeded aggregation and the amplification of pathogenic α-syn species represents a more disease-relevant and potentially effective approach for disease modification.

Despite substantial advances in understanding α-syn biology, current clinical treatments for PD and related synucleinopathies remain largely symptomatic and do not halt or reverse neurodegeneration [2]. Numerous disease-modifying strategies are under investigation, including immunotherapy, gene therapy and small-molecule approaches [6,18]. However, the structural heterogeneity of α-syn aggregates and the complexity of aggregation pathways pose significant challenges for therapeutic development [4,10,19].

Naturally occurring small molecules have emerged as attractive candidates for modulating protein aggregation due to their chemical diversity, bioactivity and favorable safety profiles. In particular, compounds derived from traditional Chinese medicine and other herbal sources have been extensively studied for their neuroprotective, anti-inflammatory and anti-aggregation properties [20]. Several such compounds have been reported to interfere with pathological protein aggregation processes relevant to neurodegenerative diseases. For example, baicalein, a flavonoid derived from *Scutellaria baicalensis*, has been shown to inhibit α-syn fibrillation, destabilize preformed fibrils and reduce α-syn–induced cellular toxicity both in vitro [21,22,23] and in vivo [24,25]. Similarly, curcumin, a major component of turmeric (*Curcuma longa*), has demonstrated inhibitory effects on amyloid aggregation and associated toxicity in multiple neurodegenerative disease models [26]. Also recent studies indicate that curcumin and its 4-arylidene derivatives can inhibit α-syn aggregation in vitro [27,28] and disaggregate the preformed fibrils [27]. Furthermore, recent work shows that plant-derived phytochemicals, particularly phenolic molecules, such as epigallocatechin-3-gallate (EGCG), resveratrol and oleuropein in addition to the above-mentioned baicalein and curcumin, exhibit anti-aggregating properties against various amyloidogenic proteins including Aβ and α-syn by modulating both the kinetics and the structure of amyloid assemblies [29]. These findings support the concept that small molecules derived from natural sources can modulate pathogenic protein aggregation pathways.

Previous studies have reported that compounds extracted from traditional Chinese medicinal herbs such as salvianolic acid B and dihydromyricetin can modulate α-syn aggregation and reduce associated toxicity [30,31], and that salvianolic acid B can interfere with amyloid formation of mutant α-syn under defined biophysical conditions. Salvianolic acid B is a major polyphenolic constituent of *Salvia miltiorrhiza* (Danshen), a traditional Chinese medicine widely used in cardiovascular indications, with well-described antioxidant and pleiotropic biological activities [32,33,34,35]. Dihydromyricetin is a flavonoid present in *Hovenia dulcis* and has been investigated for neuroactive and protective effects in several contexts [20,36,37,38,39].

However, a detailed biophysical and kinetic analysis of how these compounds affect α-syn seeded aggregation and nucleation-dependent fibrillation has remained limited.

In the present study, we systematically investigated the effects of a panel of these compounds on α-syn fibrillation and seeded aggregation. Using a combination of biochemical, biophysical and cell-based assays, we screened four compounds for their ability to interfere with α-syn aggregation and to mitigate α-syn-induced cytotoxicity. Among the compounds tested, salvianolic acid B and dihydromyricetin- emerged as particularly potent inhibitors of α-syn fibril formation and seeded nucleation, while also providing protection against α-syn–mediated cellular toxicity.

Together, these findings identify naturally derived small molecules capable of modulating key early events in α-syn aggregation and support their further exploration as potential disease-modifying agents for PD and related synucleinopathies.

## 2. Results

### 2.1. Salvianolic Acid B and Dihydromyricetin Inhibit α-Syn Fibrillation in a Concentration-Dependent Manner

**Figure 1:**
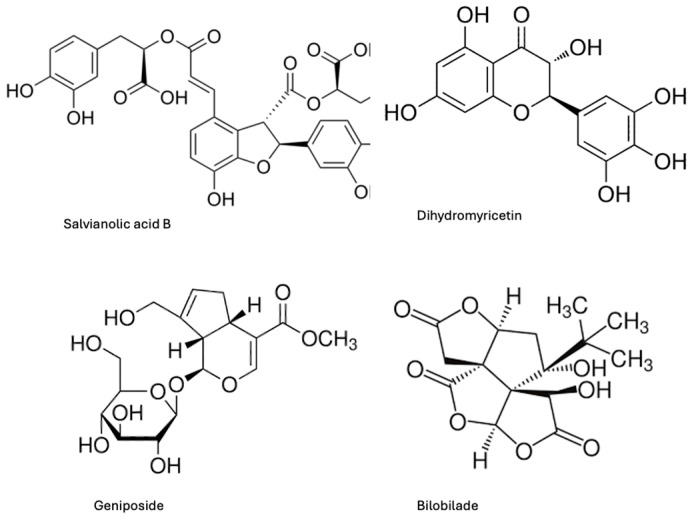
Chemical structures of naturally derived Chinese medicinal compounds used to test the effect of α-syn fibril formation. These compounds are salvianolic acid B (SAB), Dihydromyricetin (DHM), Geniposide (GEN) and Bilobilade (BIL).

**Figure 2:**
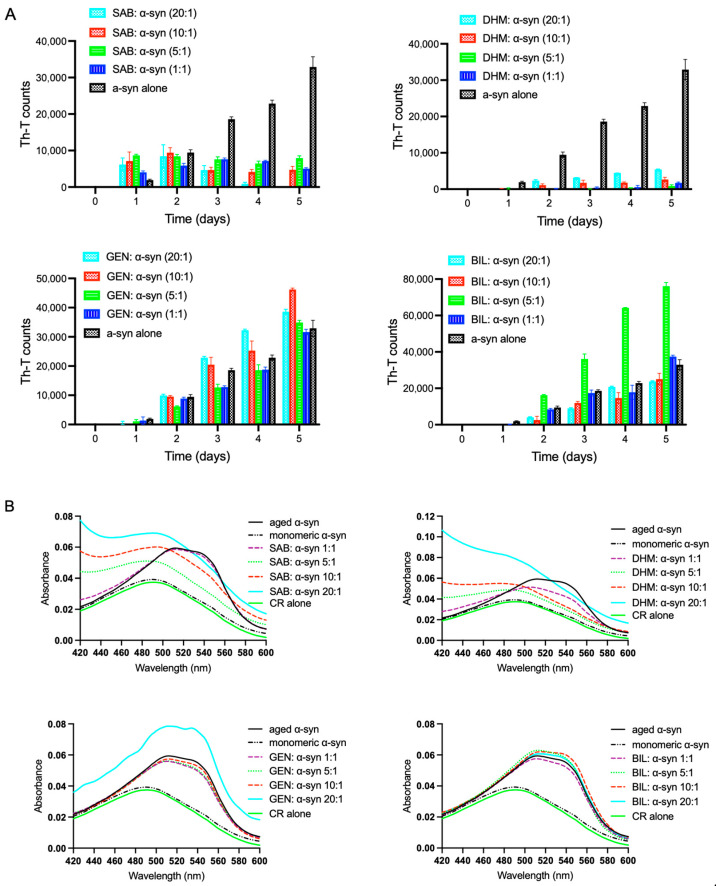

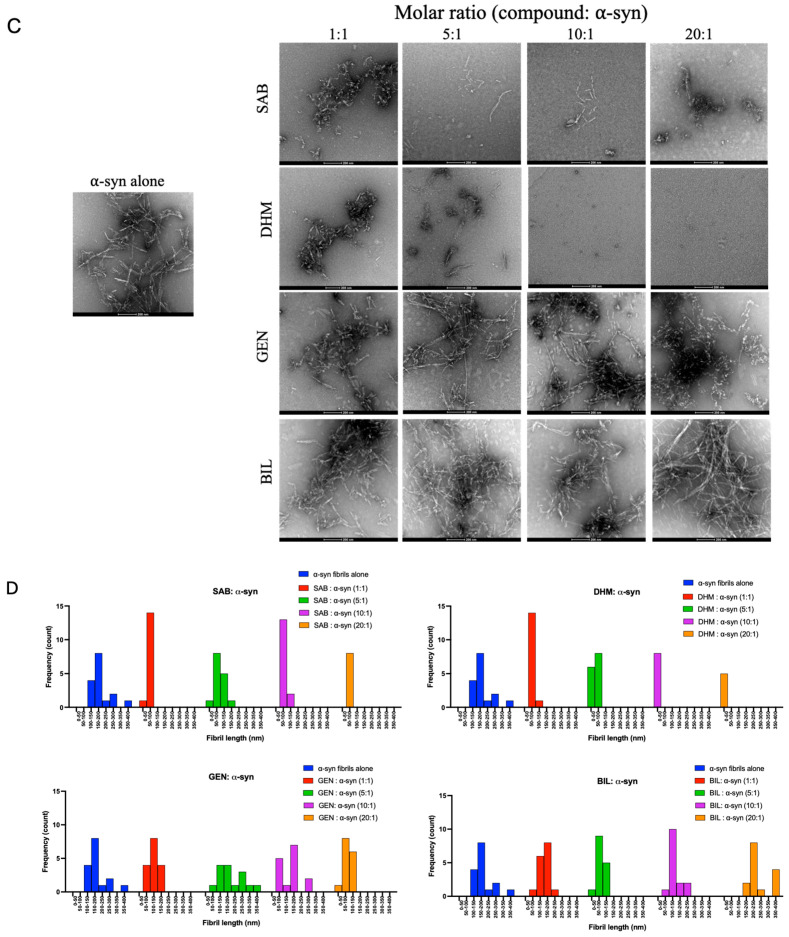
Effect of compounds on α-syn fibril formation. α-Syn protein (25 μM) was incubated for 5 days at 37 °C with continuous shaking in the absence or presence of SAB, DHM, GEN, and BIL using different molar ratios (compound: α-syn at 1:1, 5:1, 10:1 and 20:1). Fibril content for each sample was then measured using (**A**) Thioflavin-T (Th-T) and (**B**) Congo red binding assays. Means ± standard deviations are from triplicates of one experiment. (**C**) Negative stain electron microscopy images showing fibril formation of α-syn aged alone or in the presence of the indicated compounds (compound: α-syn molar ratios of 1:1, 5:1, 10:1 and 20:1). Scale bar, 200 nm. (**D**) Size distribution of α-synuclein fibrils obtained from electron microscopy images following incubation with the indicated compounds at different molar ratios (compound: α-syn). Fibril lengths were measured and the resulting distributions are presented as histograms for each condition.

To determine whether selected Chinese medicinal compounds inhibit α-syn fibril formation, four compounds, salvianolic acid B, dihydromyricetin, geniposide and bilobilade, extracted from different herbal sources, were screened (Figure 1). Fibrillation was first assessed using the Thioflavin T (Th-T) fluorescence assay (Figure 2A). Monomeric α-syn (25 μM) was incubated alone or in the presence of each compound at molar ratios of 20:1, 10:1, 5:1 and 1:1 (compound: α-syn), with continuous agitation at 37 °C for five days. Th-T fluorescence emission was measured daily to monitor fibril formation.

A pronounced reduction in Th-T fluorescence was observed in samples treated with salvianolic acid B (Figure 2A, upper left panel) and dihydromyricetin (Figure 2A, upper right panel), indicating inhibition of fibril formation relative to α-syn alone. Although both compounds exhibited inhibitory activity, dihydromyricetin was more potent. Following five days of incubation, dihydromyricetin almost completely abolished α-syn fibrillation at molar ratios of 1:1, 5:1 and 10:1, with inhibition levels of approximately 95%, 97% and 92%, respectively, while at a 20:1 molar ratio, dihydromyricetin reduced fibrillation by 84%. In contrast, salvianolic acid B achieved complete inhibition of fibrillation only at a molar ratio of 20:1, whereas inhibition at molar ratios of 1:1, 5:1, and 10:1 was approximately 85%, 76% and 86%, respectively. These findings demonstrate an inhibitory effect for both compounds, with dihydromyricetin exhibiting greater potency.

In contrast, geniposide and bilobalide displayed no significant inhibitory effect on α-syn fibrillation (Figure 2A, lower panels). Their aggregation profiles were comparable to α-syn alone, with geniposide slightly enhancing fibril formation (Figure 2A, lower left panel).

#### Congo Red Binding Confirms Inhibition of Amyloid Fibril Formation

To further assess the impact of salvianolic acid B and dihydromyricetin on amyloid formation, Congo Red (CR) binding assays were performed. CR selectively binds amyloid fibrils, producing a characteristic red shift in absorbance from approximately 490 nm to 540 nm. To exclude the possibility that the compounds interfere with the Congo red absorbance assay, we performed compound-only control measurements under identical conditions. These experiments demonstrated that none of the tested compounds altered the Congo red spectral profile or its absorbance maxima, confirming the absence of assay interference. Aged α-syn samples incubated in the absence of compounds exhibited a pronounced shift in CR absorbance maximum (Figure 2B), confirming the presence of fibrils. In contrast, samples treated with salvianolic acid B (Figure 2B, upper left panel) or dihydromyricetin (Figure 2B, upper right panel) showed absorbance maxima similar to monomeric α-syn, with wavelengths remaining below 495 nm across all concentrations tested except for salvianolic acid B at 1:1 ratio. This indicates effective suppression of amyloid fibril formation. Conversely, geniposide and bilobalide-treated samples displayed CR shifts comparable to aged α-syn alone (Figure 2B, lower panels), further confirming their inability to inhibit fibrillation. Transmission electron microscopy (TEM) provided further structural confirmation. Aged α-syn alone formed dense networks of long fibrils (Figure 2C). In contrast, samples incubated with salvianolic acid B or dihydromyricetin at all molar ratios (1:1–20:1) displayed thin, short and fragmented rod-like fibrils. Geniposide and bilobalide, by comparison, induced only minimal morphological changes even at higher concentrations (Figure 2C). These observations are further supported by the fibril length distribution analysis (Figure 2D).

### 2.2. Salvianolic Acid B and Dihydromyricetin Attenuate α-Syn-Induced Cytotoxicity

**Figure 3:**
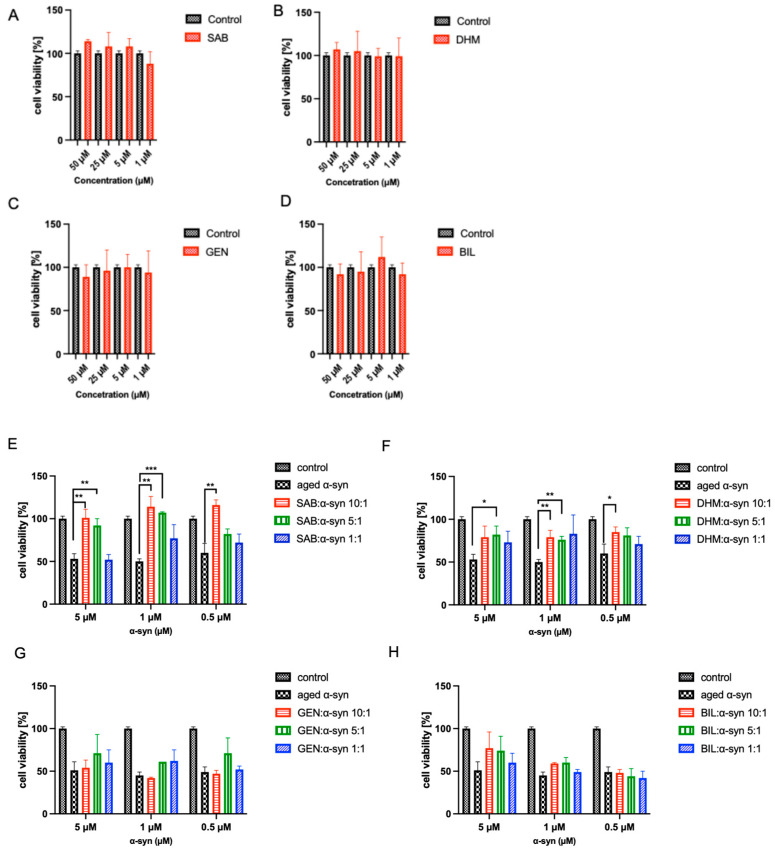
Effect of compounds alone on BE(2)-M17 cells. Cells were treated with (**A**) SAB, (**B**) DHM, (**C**) GEN, and (**D**) BIL at four different concentrations (50, 25, 5, and 1 μM) for 48 h prior to the addition of MTT. Means ± standard deviations are from the average of 4 wells. Effect of compounds on cell toxicity of α-syn aggregates. (**E**–**H**) The viability of BE(2)-M17 human cells treated with either α-syn aged alone or with (**E**) SAB, (**F**) DHM, (**G**) GEN, and (**H**) BIL was tested by MTT assay. Cells were treated with the indicated concentrations of α-syn and compounds for 48 h prior to the addition of MTT. * *p* < 0.05, ** *p* < 0.01 and *** *p* < 0.001.

Given the established link between α-syn aggregation and cellular toxicity, the effects of the compounds on α-syn-induced cytotoxicity were examined using BE(2)-M17 human neuroblastoma cells. Aged α-syn, either alone or in combination with salvianolic acid B, dihydromyricetin, geniposide, or bilobalide at compound-to-α-syn molar ratios of 10:1, 5:1, and 1:1, was added to the cells, and cell viability was subsequently assessed using the MTT assay. Cytotoxicity assays with each compound alone confirmed the absence of significant toxicity at the tested concentrations (Figure 3A–D). In contrast, aged α-syn solution alone induced a dose-dependent decrease in cell viability, with approximately 45–55% survival observed at 5 µM α-syn (Figure 3E–H). Co-incubation with salvianolic acid B significantly attenuated α-syn–induced toxicity (Figure 3E). At 5 µM α-syn, cell viability increased to approximately 100% and 90% at molar ratios of 10:1 and 5:1, respectively, with no protection observed at a 1:1 ratio. Similar protective effects were observed at lower α-syn concentrations (1 µM and 0.5 µM), where salvianolic acid B restored viability to near control levels at a 10:1 ratio. Similarly, dihydromyricetin markedly reduced α-syn–induced cytotoxicity (Figure 3F). At 5 µM α-syn, viability increased to approximately 75–85% at 10:1, 5:1 and 1:1 molar ratios. Comparable rescue effects were observed at 1 µM and 0.5 µM α-syn concentrations. In contrast, neither geniposide nor bilobalide showed significant protection against α-syn toxicity (Figure 3G,H). Cell viability in the presence of these compounds remained comparable to that observed with aged α-syn alone across all concentrations and molar ratios tested.

### 2.3. Dihydromyricetin Disaggregates Preformed α-Syn Fibrils and Attenuates Fibril-Associated Cytotoxicity

**Figure 4:**
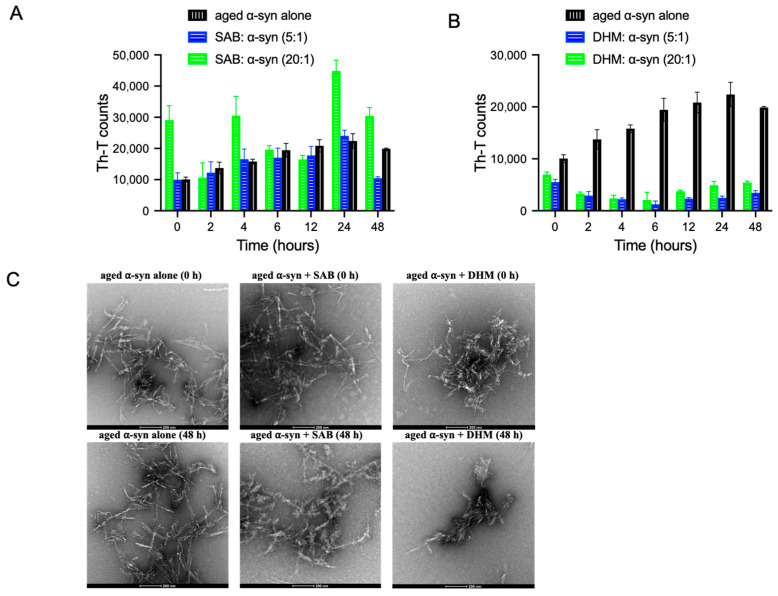
Effect of SAB and DHM on performed α-syn fibrils. (**A**,**B**) Th-T binding assay used to measure the fibril content resulted from the incubation of aggregated α-syn for the indicated times in the absence or presence of (**A**) SAB and (**B**) DHM using different molar ratios (compound: α-syn at 20:1 and 5:1). The assays were performed in triplicates (Means ± standard deviations are from the average of the triplicates). (**C**) Electron microscopy images of negatively stained samples of aged α-syn incubated alone or in the presence of SAB and DHM (compound: α-syn at 20:1) for 0 h (upper panels) or 48 h (lower panels) with continuous shaking at 37 °C. Scale bar, 200 nm.

Since both salvianolic acid B or dihydromyricetin were able to inhibit α-syn fibrillation, it was of our interest to assess whether both compounds could reverse existing fibrils. Preformed α-syn fibrils (25 μM) were incubated with salvianolic acid B or dihydromyricetin at molar ratios of 20:1 and 5:1 for 48 h. Th-T fluorescence was measured at different time points (0, 2, 4, 6, 12, 24, and 48 h) (Figure 4). In the presence of salvianolic acid B, no reduction in Th-T signal was observed (Figure 4A). At the lower molar ratio (5:1), Th-T fluorescence remained comparable to that of α-syn alone, whereas at higher ratio (20:1), salvianolic acid B led to a marked increase in Th-T signal exceeding that of α-syn alone. This suggests that salvianolic acid B did not induce disaggregation and may instead promote or stabilize Th-T-reactive β-sheet-rich aggregates or modulate Th-T binding. In contrast, dihydromyricetin induced a progressive and dose-dependent decrease in Th-T fluorescence across all time points and molar ratios tested (Figure 4B), indicating disaggregation of preformed fibrils into non-amyloid species. TEM analysis verified these findings. Long fibrils persisted in α-syn alone and salvianolic acid B-treated samples (Figure 4C, left and middle panels), whereas dihydromyricetin-treated samples displayed short, fragmented fibrils (Figure 4C, right panels). Collectively, these findings suggest that dihydromyricetin efficiently disrupts α-syn fibrils, supporting its potential as a therapeutic candidate targeting α-syn aggregation.

Its worth noting that both salvianolic acid B and dihydromyricetin effectively inhibited α-synuclein aggregation, as confirmed above by ThT fluorescence, Congo red binding, and TEM analysis (Figure 2). Consequently, the end products generated in the presence of both compounds are expected to be less toxic, which is consistent with the observed reduction in cytotoxicity in the MTT assay (Figure 3). In contrast, the disaggregation experiments represent a different condition, where pre-formed fibrils were treated with the compounds. In this setting, only dihydromyricetin showed the ability to disaggregate fibrils, whereas salvianolic acid B did not (Figure 4). Therefore, the similar cytoprotictvity observed for salvianolic acid B and dihydromyricetin (see Figure 3) reflects their shared ability to prevent the formation of the toxic fibril, rather than their disaggregation capacity.

### 2.4. Salvianolic Acid B and Dihydromyricetin Inhibit Seeded α-Syn Aggregation

**Figure 5:**
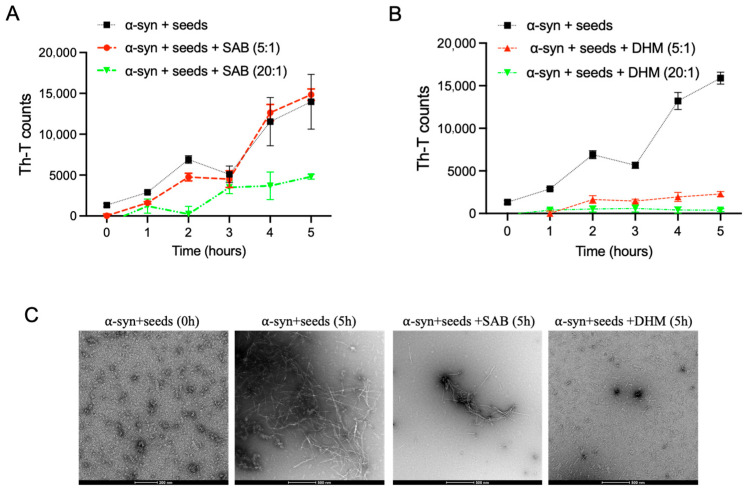
Effect of SAB and DHM on the seeding of α-syn monomers with fibrils. α-syn monomers (100 μM) were seeded with 2 μM sonicated α-syn fibrils, which were then incubated in the presence or absence of (**A**) SAB and (**B**) DHM at different concentrations (10 and 40 μM) for 5 h with continuous shaking at 37 °C. The extent of fibril formation was estimated by Th-T binding assay. The assays were performed in triplicate (average of triplicate measurements ± standard deviations). (**C**) Electron microscopy images of negatively stained samples of α-syn incubated with seeds alone or in the presence of SAB or DHM in a concentration of 50 μM for the indicated times with continuous shaking at 37 °C. Scale bar, 500 nm. d Th-T binding assay for seeding the aggregation of α-syn monomers by either 2 μM of untreated seeds (wt seeds) or seeds generated from the incubation of SAB or DHM with α-syn fibrils for 5 days at a molar ratio of 4:1.

Amyloid fibril formation proceeds through a nucleation-dependent polymerization mechanism involving three main phases: an initial lag (nucleation) phase during which soluble monomers assemble into oligomeric species, followed by a growth (polymerization) phase that leads to fibril elongation, and finally an equilibrium phase characterized by mature insoluble amyloid fibrils [40,41]. This process can be markedly accelerated by the presence of preformed fibrils or seeds, which bypass the nucleation phase and promote rapid fibril formation both in vitro and in vivo through a mechanism known as seeding [42,43,44,45]. Our above results demonstrated that both salvianolic acid B and dihydromyricetin inhibit α-syn fibril formation (Figure 2), whereas only dihydromyricetin is capable of disaggregating preformed α-syn fibrils (Figure 4). To further assess the effects of these compounds on seeded α-syn fibril growth, Th-T kinetic assays were performed in the presence of preformed α-syn seeds. Mature α-syn fibrils were sonicated to generate short fibrillar seeds, which were then added to monomeric α-syn (100 µM) at a final seed concentration of 2 µM. Aggregation assays were conducted using α-syn seeds preincubated with salvianolic acid B or dihydromyricetin at compound-to-seed molar ratios of 5:1 and 20:1 for 1 h. The reactions were then incubated with monomeric α-syn (100 µM) at 37 °C under continuous shaking for 5 h, with Th-T fluorescence measured at hourly intervals. As expected, the addition of seeds markedly accelerated α-syn fibrillation, with aggregation levels after 5 h comparable to those observed after 72 h in the absence of seeds (compare Figure 5A with Figure 2A). Notably, Salvianolic acid B failed to suppress seeded aggregation at a 5:1 molar ratio, with Th-T fluorescence increasing similarly to α-syn with seeds alone, although a moderate delay in aggregation kinetics was observed at the higher 20:1 ratio (Figure 5A). In contrast, dihydromyricetin significantly inhibited seeded fibril elongation, maintaining low Th-T fluorescence throughout the assay at both molar ratios tested (Figure 5B). These results further support that dihydromyricetin effectively blocks both spontaneous and seed-induced aggregation. To further validate these findings, TEM was performed on seeded α-syn samples incubated for 5 h in the presence or absence of each compound at a 20:1 molar ratio. In the absence of compounds, α-syn formed dense networks of long fibrils (Figure 5C). In contrast, samples containing dihydromyricetin—and to a lesser extent, salvianolic acid B - displayed only small, fragmented, rod-like structures resembling those observed at the initial time point prior to fibril formation (Figure 5C), confirming a strong inhibition of seeded fibril growth.

## 3. Discussion

PD is a progressive neurodegenerative disorder, the prevalence of which is increasing worldwide and is projected to rise substantially with global population ageing [46]. Current therapies are largely symptomatic, and there remains a major unmet need for disease-modifying interventions. A large body of evidence supports a central role for α-syn misfolding, aggregation and Lewy pathology in PD and related synucleinopathies [4,6,47,48,49]. Mechanistically, α-syn fibrillation proceeds via nucleation-dependent polymerization, during which toxic prefibrillar intermediates and fibrillar assemblies contribute to neuronal dysfunction and cell death [50,51]. Importantly, the aggregation process can be markedly accelerated by seeding, a phenomenon closely linked to the prion-like spread of pathology in vivo [15,42,43,44,52,53]. Accordingly, inhibiting aggregation and/or seed-driven propagation remains an attractive therapeutic strategy [54,55,56,57].

In the present study, we screened four bioactive natural compounds and identified salvianolic acid B and dihydromyricetin as the most active modulators of α-syn aggregation and toxicity, whereas geniposide and bilobalide showed little to no activity across assays. Salvianolic acid B is a major polyphenolic constituent of *Salvia miltiorrhiza* (Danshen), a traditional Chinese medicine widely used in cardiovascular indications, with well-described antioxidant and pleiotropic biological activities [32,33,34,35]. Dihydromyricetin is a flavonoid present in *Hovenia dulcis* and has been investigated for neuroactive and protective effects in several contexts [20,36,37,38,39].

### 3.1. Salvianolic Acid B and Dihydromyricetin Inhibit α-Syn Fibrillation but Differ in Their Effects on Preformed Fibrils and Seeding

Using complementary biochemical and structural approaches (Th-T, Congo Red, TEM), we show that both salvianolic acid B and dihydromyricetin inhibit α-syn fibrillation in vitro and alter fibril morphology, producing shorter, fragmented, rod-like structures compared with the dense fibrillar networks formed by α-syn alone. These findings are consistent with literature demonstrating that polyphenolic compounds can suppress amyloid assembly through aromatic interactions, hydrogen bonding and pathway redirection [58,59,60,61,62,63]. Prior work on related natural compounds, including baicalein, EGCG ginsenoside Rb1, oleuropein aglycone and resveratrol also supports the concept that polyphenols can inhibit α-syn aggregation and mitigate toxicity [21,64,65].

Despite comparable effects in spontaneous fibrillation assays, salvianolic acid B and dihydromyricetin diverged markedly in their ability to target preformed fibrils and seeded aggregation. Dihydromyricetin produced a progressive decrease in Th-T fluorescence when incubated with preformed fibrils and caused clear fibril fragmentation by TEM, supporting a true fibril-destabilizing/disaggregating activity, similar to other anti-amyloid polyphenols reported to destabilize amyloid assemblies [60]. In contrast, salvianolic acid B did not reduce Th-T signal from preformed fibrils and at high concentrations increased Th-T fluorescence beyond α-syn alone. This pattern suggests that salvianolic acid B does not disaggregate mature fibrils and may instead stabilize Th-T-reactive β-sheet-rich conformers and/or alter Th-T binding properties. Such dye-related effects are well recognized, as Th-T fluorescence depends on binding mode and fibril polymorphism and can be modulated by small molecules even without proportional changes in aggregate mass [66].

In seeded aggregation assays, dihydromyricetin potently inhibited templated fibril elongation at both ratios tested, maintaining low Th-T fluorescence throughout the assay. Given the established relevance of seeded propagation to the initiation and spreading of α-syn pathology in vivo, this property is particularly significant [44,45]. Salvianolic acid B, however, showed limited suppression of seeded aggregation, with an evident effect only as a kinetic delay at the higher ratio. This suggests that salvianolic acid B is less effective at blocking fibril-end elongation or templated conversion under strongly seeded conditions. Nevertheless, TEM analysis at the end of the seeded assay indicated that, at higher concentration, salvianolic acid B limited the formation of extensive mature fibril networks, consistent with an ability to perturb growth trajectories or promote shorter/fragmented fibrillar structures. Together, these data point to dihydromyricetin as a robust inhibitor of both spontaneous and seed-driven aggregation, whereas salvianolic acid B appears to act primarily as an aggregation modulator.

### 3.2. Cytoprotective Effect of Salvianolic Acid B and Dihydromyricetin

Both salvianolic acid B and dihydromyricetin significantly reduced α-syn–induced cytotoxicity in BE(2)-M17 neuroblastoma cells, whereas geniposide and bilobalide did not confer meaningful protection. The protective effect of dihydromyricetin is consistent with its strong suppression of fibril formation, inhibition of seeded elongation and disaggregation of mature fibrils. Salvianolic acid B, in contrast, reduced toxicity despite its limited effects on preformed fibrils and seeded aggregation, suggesting that it may detoxify early aggregation intermediates or redirect α-syn into less harmful assemblies. This interpretation is consistent with the concept that soluble oligomeric species can be major drivers of toxicity and that different aggregate conformations may differ markedly in their pathogenicity [67,68,69]. In addition, several polyphenols have been shown to remodel amyloidogenic proteins into off-pathway or less structured oligomers with reduced toxicity [61,70,71], supporting a plausible framework for salvianolic acid B-mediated cytoprotection even when Th-T-reactive assemblies persist.

The primary aim of our work was to evaluate the effects of salvianolic acid B and dihydromyricetin on α-synuclein aggregation and associated cytotoxicity. However, our data provide useful mechanistic insights into their distinct modes of action. Specifically, our results suggest that dihydromyricetin primarily interacts with α-syn species in a manner that promotes disaggregation of preformed fibrils, whereas salvianolic B does not exhibit disaggregation activity but effectively inhibits aggregation and reduces toxicity. Based on these observations, we propose that dihydromyricetin may act on later-stage aggregated species (e.g., fibrils), while salvianolic acid B may preferentially interact with earlier species in the aggregation pathway, such as monomers or intermediate aggregates, thereby preventing their progression into toxic assemblies. This differential behavior may explain why both compounds reduce cytotoxicity despite their distinct effects on fibril disaggregation. Previous studies utilizing molecular dynamics simulations revealed that dihydromyricetin interacts with the α-syn trimer mainly via nonpolar mechanisms, with key hydrophobic residues whose side chains and main chains exhibit a synergistic effect through hydrophobic and hydrogen-bonding interactions [30], while studies on α-syn A53T using computational and experimental approaches have demonstrated that salvianolic acid B effectively prevents the amyloid fibrillation of A53T α-syn monomers by interacting with the N-terminal region and NAC domain, particularly associating with the KTKEGV motif and NAC segment through hydrophobic and hydrogen bonding interactions [31].

While these findings clarify aspects of aggregate remodelling and toxicity, the precise molecular pathways and targets through which salvianolic acid B and dihydromyricetin exert their effects remain to be determined.

### 3.3. Limitations and Future Directions

Although we used multiple orthogonal readouts (Th-T, Congo Red, TEM, and toxicity), certain limitations remain. Dye-based assays can be influenced by compound-specific effects on probe binding and fluorescence, which is particularly relevant to interpreting the increased Th-T signal seen with salvianolic acid B at high ratios in the disaggregation assay [66]. Additional orthogonal approaches, such as sedimentation/insoluble fraction quantification and conformer-specific immunodetection, would further strengthen conclusions about aggregate load versus dye binding. Moreover, defining which α-syn species are most affected (toxic oligomers vs. benign assemblies) will benefit from conformational and oligomer-selective tools and structural analyses. While both salvianolic acid B and dihydromyricetin demonstrated significant effects on α-syn aggregation and associated cytotoxicity in our in vitro models, the concentrations used in this study may exceed those achievable under physiological conditions. Therefore, these findings should be interpreted primarily in a mechanistic context rather than as direct evidence of therapeutic potential. The observed effects provide insight into how these compounds differentially modulate α-syn species and toxicity, but do not establish their efficacy in vivo. Further studies will be required to evaluate their pharmacokinetic properties, bioavailability, and activity at physiologically relevant concentrations. Such investigations will be essential to determine the extent to which these in vitro observations translate to more complex biological systems. Finally, in vivo evaluation will be critical, as seeded transmission initiates Parkinson-like neurodegeneration in animal models [45], and the pharmacokinetics, brain exposure, and safety profiles of these compounds will determine their translational potential.

### 3.4. Conclusions

In conclusion, salvianolic acid B and dihydromyricetin were the most effective modulators of α-syn aggregation. Dihydromyricetin inhibits α-syn aggregation and even promotes fibril disaggregation, while salvianolic acid B only reduces the aggregation process of α-syn, likely by modulating toxic intermediates. Together, these compounds represent promising leads for further mechanistic studies in synucleinopathy models. Future studies will be required to assess the pharmacokinetic properties, bioavailability, and efficacy of these compounds at physiologically relevant concentrations.

## 4. Materials and Methods

### 4.1. Expression and Purification of Recombinant Human α-Syn

Full-length recombinant human α-syn was expressed in *Escherichia coli* BL21 (DE3) using the bacterial expression vector pRK172. Following expression and sedimentation, the bacterial pellets from 1 L of TB broth were homogenized and sonicated in 50 mL of high-salt buffer (0.75 M NaCl, 10 mM Tris, pH 7.6, 1 mM EDTA) containing a cocktail of protease inhibitors (Thermo Scientific, Waltham, MA, USA), heated to 100 °C for 10 min and centrifuged at 5300× *g* for 20 min. The solution was dialyzed overnight against the buffer used for gel filtration chromatography (50 mM NaCl, 10 mM Tris, pH 7.6, 1 mM EDTA), following which, the volume was reduced to 5 mL using a Pierce protein concentrator (10 K MWCO; ThermoFisher Scientific, Waltham, MA, USA) according to the manufacturer’s instructions. All proteins were purified by size exclusion using a Superdex 200 gel filtration column (GE Healthcare, Chicago, IL, USA). The clean fractions were pooled, exchanged with a buffer (10 mM Tris pH 7.6, 25 mM NaCl, 1 mM EDTA, 1 mM PMSF) for ion exchange chromatography by dialysis overnight, and were applied onto a HiTrap Q column (GE Healthcare, Chicago, IL, USA) and eluted in 10 mM Tris pH 7.6 using a linear gradient of 0.025–1.0 M NaCl. Purified fractions were pooled, and protein concentrations were determined using the Pierce BCA protein assay kit (ThermoFisher Scientific).

### 4.2. In Vitro α-Syn Aggregation Assays

α-Syn aggregation was performed under controlled conditions in the presence or absence of tested compounds. Stock solutions of the compounds (Salvianolic acid B, Dihydromyricetin, Geniposide and Bilobalide; Sigma-Aldrich St. Louis, MO, USA and Burlington, MA, USA) were prepared at 10 mM in dimethyl sulfoxide (DMSO). α-Syn was diluted in phosphate-buffered saline (PBS) to a final concentration of 25 μM and incubated with each compound at molar ratios of 20:1, 10:1, 5:1 or 1:1 (compound: α-syn). The concentrations used in this study were selected based on (i) previously published in vitro studies demonstrating activity against α-synuclein aggregation, and (ii) preliminary dose–response experiments performed in our laboratory to identify concentrations that are biologically effective while remaining non-toxic to cells. These ranges were chosen to ensure sufficient activity in aggregation/disaggregation assays without introducing compound-related cytotoxicity. The final DMSO concentration was maintained at 1% (*v*/*v*) in all samples. Samples were incubated at 37 °C for up to 5 days with continuous agitation at 800 rpm using a Thermomixer (Eppendorf, Hamburg, Germany). Aliquots were collected at defined time points for downstream analyses.

### 4.3. Thioflavin-T Fluorescence Assay

Amyloid fibril formation was monitored using the Thioflavin-T (Th-T) binding assay. At each time point, 10 μL of α-syn sample was mixed with 40 μL of Th-T stock solution to achieve final concentrations of 5 μM α-syn and 20 μM Th-T. Fluorescence measurements were performed using a Victor X3 microplate reader (PerkinElmer, Waltham, MA (Massachusetts), Boston, MA, or Shelton, CT (Connecticut), USA) with excitation at 450 nm and emission at 486 nm, using black 384-well plates (Nunc, Roskilde, Denmark).

### 4.4. Transmission Electron Microscopy (TEM)

Morphological characterization of α-syn aggregates was performed by transmission electron microscopy. α-Syn samples aged in the presence or absence of the compounds were applied to 400-mesh copper grids (Agar Scientific, Smethurst High-Light Ltd. (Bolton, UK)) and allowed to adsorb. Samples were fixed with 0.5% glutaraldehyde and negatively stained with 2% uranyl acetate. Imaging was performed using a Talos F200X transmission electron microscope (Thermo Fisher Scientific), operated at 200 kV.

### 4.5. Congo Red Binding Assay

Congo red binding was used as an independent assessment of amyloid formation. Compounds alone or five-day-aged α-syn samples with or without compounds were incubated with 20 μM Congo red prepared in PBS and filtered through a 0.45 μm membrane. Absorbance spectra between 400 and 600 nm were recorded using 10 mm quartz cuvette by a DU-800 UV–Vis spectrophotometer (Beckman Coulter, CA, USA).

### 4.6. Cell Culture and Viability Assays

Human BE(2)-M17 neuroblastoma cells were maintained in Dulbecco’s MEM/Nutrient Mix F-12 (1:1) (Gibco BRL, Rockville, MD, USA) supplemented with 15% fetal bovine serum and 1% penicillin–streptomycin (Sigma-Aldrich) at 37 °C in a 5% CO_2_/95% air humidified incubator to maintain their growth.

Measurement of cell viability—A cell density of 15,000 per well, were plated in a 96-well plate in DMEM medium. The cells were cultured for 24 h before replacing the media with 200 μL of OPTI-MEM (Gibco- Grand Island, NY, USA) serum-free medium containing aged α-syn solutions with or without the compounds. The cells were then incubated for 48 h (37 °C, 5% CO_2)_. To conduct the MTT assay, 20 μL per well of MTT (3-(4, 5-dimethylthiazol-2-yl)-2,5-diphenyltetrazolium bromide) (Sigma-Aldrich, USA) at a concentration of 6 mg/mL in PBS, was added to the cells, followed by 4.5 h incubation at 37 °C. The medium was discarded carefully, and the cells were then lysed by incubating at 37 °C overnight in 100 μL of lysis buffer (15% SDS, 50% N,N-dimethylformamide, pH 4.7). Absorbance at 590 nm was measured by the microplate reader victor X3 (PerkinElmer, Waltham, MA, Boston, MA, or Shelton, CT, USA).

### 4.7. Seeded Polymerization Assay

Monomeric α-syn (100 µM) was aggregated by incubating at 37 °C for up to 7 days with continuous agitation at 800 rpm using a Thermomixer (Eppendorf). The crude α-syn fibril sample was spun at 10,000× *g* for 10 min at 4 °C in a refrigerated microfuge (Eppendorf). The supernatant was then discarded, and the pellet was washed twice and finally resuspended in 1XPBS to get pure fibrils. The pure fibrils were fragmented by ultrasonication keeping on ice using a Sonic ruptor 250, equipped with a fine tip (2 s pulses, output of 40 watts for 5 min) to get pure “seeds”. To determine the concentration of seeds, the samples were denatured with equal volume of 6 M Guanidine-HCl and quantified using Pierce BCA protein assay kit (ThermoFisher Scientific)

For the seeded polymerization assay, 2 μM of seeds were incubated for one hour in the presence or absence of compounds followed by addition to 100 μM monomeric α-syn. The solutions were incubated at 37 °C for 5 h with continuous shaking at 800 rpm. The fibril formation in α-syn samples were monitored by Th-T binding assay as described above.

### 4.8. Disaggregation Assay

As described above, preformed α-syn fibrils (25 μM) were incubated with compounds at molar ratios of 5: 1 or 20:1 (compounds: α-syn) for 48 h at 37 °C with continuous mixing at 800 rpm. The fibril content was assessed by Th-T assay.

## Data Availability

The raw data supporting the conclusions of this article will be made available by the authors on request.

## Abbreviations

The following abbreviations are used in this manuscript:

PD	Parkinson’s disease
LBs	Lewy bodies
LNs	Lewy neurites
EDTA	Ethylenediaminetetraacetic acid
DMSO	Dimethyl sulfoxide
PMSF	Phenylmethyl sulfonyl fluoride
SAB	Salvianolic acid B
DHM	Dihydromyricetin
GEN	Geniposide
BIL	Biobalide
TEM	Transmission electron microscopy
